# Supplementary material for: Analysis of PPARγ Signaling Activity in Psoriasis
Source: Int J Mol Sci. 2021 Aug 10;22(16):8603. doi: 10.3390/ijms22168603 (PMC8395241; doi:10.3390/ijms22168603)
Supplement: Supplementary file 1 [file ijms-22-08603-s001.zip › Supplemental materials_Analysis of PPARg signaling activity in psoriasis/Pathway models/Models images and html files/Model of PPARG signaling in psoriasis/1360.html]

CXCR5


# Protein CXCR5

|  |  |
| --- | --- |
| URN | urn:agi-llid:643 |
| Total Entities | 0 |
| Connectivity | 869 |
| Name | CXCR5 |
| Description | C-X-C motif chemokine receptor 5 |
| Notes | This gene encodes a multi-pass membrane protein that belongs to the CXC chemokine receptor family. It is expressed in mature B-cells and Burkitt's lymphoma. This cytokine receptor binds to B-lymphocyte chemoattractant (BLC), and is involved in B-cell migration into B-cell follicles of spleen and Peyer patches. Alternatively spliced transcript variants encoding different isoforms have been described for this gene. [provided by RefSeq, Aug 2011] |
| Primary Cell Localization | Plasma membrane |
| Class | Receptor |

---

|  |  |
| --- | --- |
| Pathway | CXC Chemokine Receptor Signaling |
|  | CD16/CD14 Proinflammatory Monocyte Activation |
|  | CD8+ Naive T-cell -> CD4+ Naive T-cell Surface Expression Markers |
|  | T-lymphoid precursor cell -> CD8+ Naive T-cell Surface Expression Markers |
|  | Thymic Follicular Hyperplasia |
|  | Proteins Involved in B-Cell Chronic Lymphocytic Leukemia |
|  | B-Cell Chronic Lymphocytic Leukemia |
|  | Follicular Lymphoma |
|  | Mantle Cell Lymphoma |
|  | Proteins Involved in T-Cell Acute Lymphoblastic Leukemia |
|  | Proteins Involved in Neuroblastoma |
|  | Elevated Receptors -> Expression Targets in Lymph Node |
|  | CD Markers -> Expression Targets in Lymphoid System and Blood |
|  | Chemokine Receptor Family -> Expression Targets in Lymphoid System |
|  | Model of PPARG signaling in psoriasis |

---

|  |  |
| --- | --- |
| MedScan ID | 643 |

---

|  |  |
| --- | --- |
| LocusLink ID | 643 |
|  | 12145 |
|  | 29363 |

---

|  |  |
| --- | --- |
| Alias | CCR5\_HUMAN |
|  | CD185 |
|  | MDR15 |
|  | chemokine (C-X-C motif) receptor 5 |
|  | Burkitt lymphoma receptor 1 isoform II |
|  | Burkitt lymphoma receptor 1 isoform 2 |
|  | Burkitt lymphoma receptor 1, GTP binding protein |
|  | Burkitt lymphoma receptor 1 isoform 1 |
|  | Burkitt'S lymphoma receptor I |
|  | neurolymphatic receptor |
|  | Blr I |
|  | C-X-C chemokine receptor type 5 |
|  | CXCL13 receptor |
|  | chemokine receptor CXCR5 |
|  | chemokine receptors CXCR5 |
|  | Burkitt lymphoma receptor 1, GTP binding protein (chemokine (C-X-C motif) receptor 5) |
|  | antigen CD185 |
|  | burkitt lymphoma receptor 1 homolog |
|  | CXCR5 receptor |
|  | CXCR5 receptors |
|  | G protein-coupled receptor BLR1 |
|  | C-X-C chemokine receptor type V |
|  | LOC112887 |
|  | LOC115271 |
|  | CD185 antigen |
|  | MGC117347 |
|  | CXCL13 receptors |
|  | Burkitt lymphoma receptor 1 |
|  | Monocyte-derived receptor 15 |
|  | CXC-R5 |
|  | BLC receptor |
|  | C-X-C motif chemokine receptor 5 |
|  | Blr1 |
|  | chemochine (C-X-C motif) receptor 5 |
|  | Burkitt lymphoma receptor I |
|  | G protein-coupled receptor BLR I |
|  | CCR5\_MOUSE |
|  | CXCR5 |
|  | Burkitt'S lymphoma receptor 1 |
|  | B-lymphocyte chemoattractant receptor |
|  | Burkitt lymphoma receptor 1, GTP-binding protein |
|  | CXCR-5 |
|  | MDR-15 |
|  | Gpcr6 |
|  | NLR |

---

|  |  |
| --- | --- |
| GO ID | 0019957 |
|  | 0016493 |
|  | 0016494 |
|  | 0004930 |
|  | 0019956 |
|  | 0042113 |
|  | 0007186 |
|  | 0019722 |
|  | 0060326 |
|  | 0070098 |
|  | 0006935 |
|  | 0006955 |
|  | 0030595 |
|  | 0048535 |
|  | 0032467 |
|  | 0007204 |
|  | 0009897 |
|  | 0005887 |
|  | 0005886 |
|  | 0007165 |
|  | 0016021 |
|  | 0016020 |

---

|  |  |
| --- | --- |
| KEGG ID | hsa:643 |
|  | mmu:12145 |
|  | rno:29363 |

---

|  |  |
| --- | --- |
| Organism | Homo sapiens {Organism urn:agi-taxid:9606} |
|  | Mus musculus {Organism urn:agi-taxid:10090} |
|  | Rattus norvegicus {Organism urn:agi-taxid:10116} |

---

|  |  |
| --- | --- |
| Mouse chromosome position | 9 24.84 cM |

---

|  |  |
| --- | --- |
| OMIM ID | 601613 |

---

|  |  |
| --- | --- |
| Rat chromosome position | 8q22 |

---

|  |  |
| --- | --- |
| Hugo ID | 1060 |

---

|  |  |
| --- | --- |
| Human chromosome position | 11q23.3 |

---

|  |  |
| --- | --- |
| Swiss-Prot Accession | A0N0R2 |
|  | A8K647 |
|  | P32302 |
|  | Q2YD84 |
|  | P32302.1 |
|  | Q04683 |
|  | Q04683.2 |
|  | P34997 |
|  | G3V7M1 |
|  | P34997.1 |
|  | Q14811 |
|  | Q6P3C2 |

---

|  |  |
| --- | --- |
| PIR ID | S26667 |
|  | S42628 |
|  | S32785 |

---

|  |  |
| --- | --- |
| GenBank ID | NC\_000011 |
|  | NM\_001716 |
|  | NP\_001707 |
|  | NM\_032966 |
|  | NP\_116743 |
|  | AP004609 |
|  | CH471065 |
|  | EAW67415 |
|  | EAW67416 |
|  | EF064770 |
|  | ABK41953 |
|  | EF444957 |
|  | ACA05956 |
|  | JC535580 |
|  | CEF39387 |
|  | X83755 |
|  | CAA58713 |
|  | AK291512 |
|  | BAF84201 |
|  | AW575330 |
|  | BC110352 |
|  | AAI10353 |
|  | CO958128 |
|  | DA815880 |
|  | X68149 |
|  | CAA48252 |
|  | X68829 |
|  | CAA48723 |
|  | P32302 |
|  | NC\_000075 |
|  | NM\_007551 |
|  | NP\_031577 |
|  | AC125129 |
|  | CH466522 |
|  | EDL25602 |
|  | JC535582 |
|  | CEF39388 |
|  | X83756 |
|  | CAA58714 |
|  | AK089611 |
|  | AK133064 |
|  | BAE21492 |
|  | AK155954 |
|  | BAE33519 |
|  | AK156087 |
|  | BAE33583 |
|  | AK157016 |
|  | BAE33932 |
|  | BC064059 |
|  | AAH64059 |
|  | L20332 |
|  | AAA16852 |
|  | X71788 |
|  | CAA50673 |
|  | Q04683 |
|  | NC\_005107 |
|  | XM\_006242901 |
|  | XP\_006242963 |
|  | NM\_053303 |
|  | NP\_445755 |
|  | AC\_000076 |
|  | AAHX01054361 |
|  | AC105645 |
|  | CH473975 |
|  | EDL95327 |
|  | X71463 |
|  | CAA50582 |
|  | P34997 |

---

|  |  |
| --- | --- |
| Swiss-Prot ID | CXCR5\_HUMAN |
|  | CXCR5\_MOUSE |
|  | CXCR5\_RAT |

---

|  |  |
| --- | --- |
| Cell Localization | Cell membrane |

---

|  |  |
| --- | --- |
| Ensembl ID | ENSG00000160683 |
|  | ENSP00000292174.4 |
|  | ENST00000292174.4 |
|  | ENSMUSG00000047880 |
|  | ENSMUSP00000050444.7 |
|  | ENSMUST00000062215.7 |
|  | ENSRNOG00000012430 |
|  | ENSRNOP00000016580.1 |
|  | ENSRNOT00000016580.3 |

---

|  |  |
| --- | --- |
| MGI ID | MGI:103567 |

---

|  |  |
| --- | --- |
| RGD ID | 62010 |

---
